# Self-offloading therapeutic footwear using compliant snap-through arches

**DOI:** 10.1017/wtc.2022.2

**Published:** 2022-05-10

**Authors:** Priyabrata Maharana, Jyoti Sonawane, Pavan Belehalli, Gondi Kondaiah Ananthasuresh

**Affiliations:** 1 Mechanical Engineering, Indian Institute of Science, Bengaluru, India; 2 Department of Podiatry, Karnataka Institute of Endocrinology and Research, Bengaluru, India

**Keywords:** critical dynamic force, offloading, plantar pressure, snap-through, switchback time

## Abstract

In diabetic peripheral neuropathy, offloading high-plantar-pressure areas using *statically offloaded* customized insoles or expensive sensors and actuators are commonly-followed treatment procedures. In this article, we propose the concept of *dynamically self-offloading* therapeutic footwear that operates mechanically without using sensors and actuators. We achieve this by using an array of snapping arches. When a load higher than a bespoke value is applied, these arches enter negative-stiffness regime and offload the high-pressure region by snapping to a different shape. They again return to their initial shape when the load disappears. Thus, they serve as both sensors and actuators that get actuated by person’s body weight. We present an analytical method to compute the switching load and the switchback time of such arches and use them to customize the footwear according to the person’s body weight, gait speed, and foot size. We identify the high-pressure regions from the clinical data and place the arches such that these high-pressure regions get dynamically offloaded, and the pressure gets redistributed to other regions. We considered 200 kPa as a limiting pressure to prevent the prolonged effects of high plantar pressure. To check the efficacy of the concept, a complete 3D-printed prototype made of thermoplastic polyurethane was tested and compared with barefoot and in-shoe plantar pressure for subjects recruited at a clinical facility. We notice that the self-offloading insole shows the plantar pressure reduction at all the foot regions, and significant offloading of 57% is observed at the forefoot region.

## Introduction

Peripheral neuropathy is one of the most common and long-term complications of diabetes mellitus. The damage to nerves leads to loss of protective sensations, due to which any pain or foot injuries may remain unnoticed, leading to ulcers and amputation if left untreated (Duckworth et al., 1985; Cavanagh and Bus, 2010). Elevated plantar pressure is also one of the factors causing skin breakdown, foot injuries, and subsequently resulting in foot-ulceration (Mueller, 1999). Various studies linked the onset of neuropathy with elevated plantar pressure as a major contributor to ulceration (Veves et al., 1992; Payne et al., 2002). There are several reports of multiple methods for the prevention and treatment of ulceration in diabetics. It is clinically proven that correctly identifying and offloading the high-plantar-pressure regions provides relief to diabetics (Bus and Valk, 2008; Ledoux et al., 2013) and is considered the most effective way of ulcer prevention (Bus, 2012).

**Figure 1.**
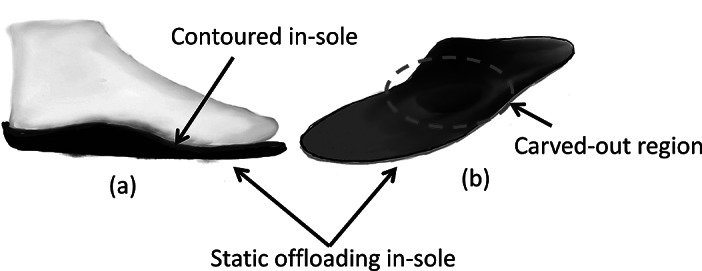
Static offloading in-sole used in current practice, (a) a contoured insole for even distribution of pressure, (b) in-sole with a carved-out region if there is an ulcer.

Various intervention techniques such as casting, surgery, custom-made orthoses, and therapeutic footwears are widely used to manage diabetic foot ulcer (DFU) or a pre-ulcerative lesion (Cavanagh and Bus, 2010; Bus et al., 2020a, b). In clinical practice, footwear offloading remains one of the routinely used techniques. Custom-molded orthoses showed a significant reduction of peak plantar pressure by increasing the contact area (Kato et al., 1996) between the foot and the insole. Modifications to the footwear by customizing the insole help in reducing the pressure in at-risk areas by redistributing it to other areas of the foot (Kato et al., 1996; Bus et al., 2004, 2011; Arts et al., 2012). Further enhancement in offloading can also be achieved by using insoles of different materials or by shaping them according to the person’s pressure contours (Viswanathan et al., 2004; Owings et al., 2008). One of the custom insoles is shown in Figure 1a,b. This is called the *static* offloading insole. This type of insole is custom-made at foot clinics. The barefoot plantar pressure measurements along with foam impressions of the patient’s foot are taken, and the locations of the ulcers or high-pressure regions are identified. A pocket is carved out, or a contour is created at identified regions in the insole so that those regions are offloaded fully or partially. However, this type of offloading is not appropriate for changing gait of a patient. Over time, the load gets transferred to the other areas of the plantar surface and would result in high-pressure points. This makes the *static* offloading footwear less effective unless it is changed periodically. Thus, the chances of DFUs reappearing with recurrent ulcers at the same or a different place in the foot are very high. The constant change in the pressure distribution of different areas of a human foot during a normal gait cycle with multiple contributing factors (i.e., anatomic changes or diseases affecting the muscles, bones, ligaments or nerves, factors involving the type of footwear, and body mass index [BMI]) make it almost impossible to prescribe a perfectly offloading footwear that serves well for a long duration.

Hence, customized insoles need frequent modification to take care of changes in pressure distribution. It is seen that patients with late-stage neuropathy need to change their offloading footwear every 4–6 months, sometimes even more frequently. This makes static offloading costly and time-consuming. Noncompliance of patients to report back to the clinic is a serious issue in follow-up treatment. Furthermore, as the contact time of foot with the ground in neuropathic patients is longer than in normal subjects, plantar pressure varies greatly during the gait cycle (heel strike, flat foot, mid stance, and heel off) for patients with diabetic neuropathy (Bacarin et al., 2009). These overstressed regions vary during the gait cycle, and hence *dynamic offloading* is beneficial to take care of these changing high-pressure regions. In other words, as the high-pressure region changes, the footwear or the customized insole should automatically adapt to the changes. Grivon et al. (2015) presented one such concept of controllable variable stiffness sole to account for dynamic pressure changes. This sole has sensors and actuators with integrated electronics. Even though this is a workable concept, the inclusion of sensors and electronic parts will need more care and maintenance than an entirely mechanically operated self-offloading insole. The use of sensors makes it expensive too. Therefore, we conceived a concept that relies on neither sensors and actuators nor electronics but provides customizable dynamic and adaptive self-offloading using only mechanically snapping arches.

In this article, we focus on designing and customizing an array of arches that serve as dynamic self-offloading footwear based on a person’s body weight, walking speed, and foot size. To offload the high-pressure regions, we use shallow arches with fixed–fixed boundary conditions. The mechanism of dynamic self-offloading footwear is explained in section “Mechanically Self-Offloading Footwear.” These kinds of arches snap at a predetermined threshold load and switch back to their original position once the load is removed. In this article, we present closed-form expressions for the switching load and switchback time of such arches and use them to customize the proposed footwear. In order to design the self-offloading footwear, we must know the pressure distribution to decide the threshold load for the arches. Therefore, we carried out plantar pressure measurements for the subjects recruited for this work (human ethical clearance obtained) and studied the pressure distribution for 10 anatomical regions of the foot, details of which are covered in section “Materials and Methods.” We discuss the design process of arches in section “Embodiment Design.” The concept is demonstrated using a 3D-printed prototype. The detailed outcomes of mechanical and clinical testing of the prototype are discussed in section “Prototyping and Testing of Self-Offloading Footwear,” followed by the concluding remarks.

## Mechanically Self-Offloading Footwear

**Figure 2.**
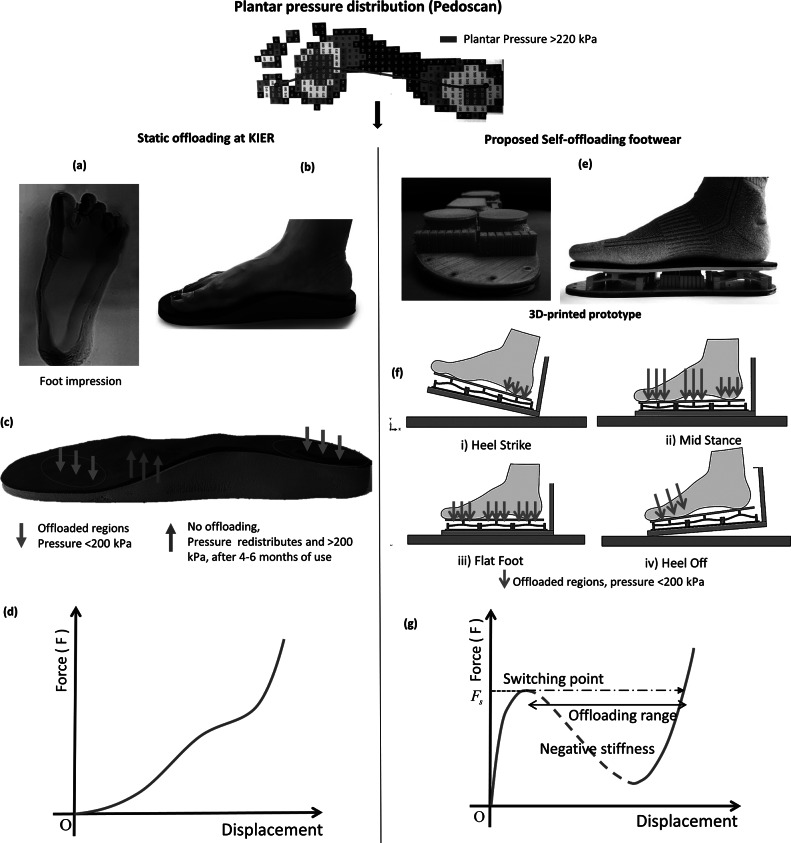
Comparison of static offloading with dynamic self-offloading. (a) Pedoscan showing plantar pressure distribution; (b) custom-carved static offloading insole from the imprint of the foot; (c) offloading of pressure in static footwear; it may be noted that another portion is now excessively loaded; (d) force-displacement curve of static offloading footwear showing positive stiffness throughout, and hence increasing force; (e) novel self-offloading footwear demonstrated using a 3D-printed prototype; (f) snapping of an array of arches for given gait cycle phase represented through simulation; (g) forcedisplacement curve for self-offloading footwear with the negative-stiffness and offloading regions marked.

The concept of mechanical self-offloading footwear is depicted in Figure 2, where the static offloading is contrasted with dynamic offloading. Both approaches begin with the plantar pressure distribution measured from a Pedoscan device, as shown in Figure 2, in which the red color indicates regions with more than 200 kPa that need to be offloaded.

In static offloading, a negative imprint of the foot is taken, and the contour of the foot is captured. The plantar pressure distribution and the foot contour are used to shape a block of phenolic foam such that the high-pressure regions and the ulceration area get offloaded (see Figure 2b). As shown in Figure 2c, the heel and the MTH (Metatarsal Head) regions get offloaded, whereas the midfoot region takes the maximum percentage of the body weight. Although this type of offloading gives relief to the patient in the initial phase, the midfoot area becomes vulnerable gradually due to the continuous excessive loading. This is not desirable. Also, note that, as shown in Figure 2d, most foam materials have a nonlinear but positive stiffness throughout the deformation, that is, the reaction force continuously increases with the deformation of the foam. This is where our concept differs.

Now, consider the dynamic self-offloading concept with snapping arches as shown in Figure 2e. Multiple arrays of fixed–fixed arches are arranged to act as an insole. A rigid capping plate attached to an array of arches supports a region of the sole of the foot. Each array has a certain threshold force beyond which it snaps to its inverted shape. As shown in Figure 2f, when the force at the heel area exceeds the threshold value during the heel strike, the array of arches underneath inverts its shape. It causes the capping plate to lose contact with the heel region momentarily, thus offloading that region. During another part of the gait cycle, the snapped arches would move up to support the load partially. Thus, the different arrays of arches redistribute the load throughout the gait cycle ensuring that the plantar pressure does not exceed the prescribed value anywhere in the sole at any time. The important aspect of the snapping arch is its changing stiffness, as shown in Figure 2g. There is a range in the force-displacement curve of the arch where it has a negative-stiffness regime (marked with a dashed line in Figure 2g). As the force on the arch exceeds the threshold value, it enters this negative force-displacement slope regime. As the negative-stiffness regime is unstable, the arch will bypass the negative as well as lower-stiffness regions to snap instantly, thereby losing contact with the sole. However, the inverted arch will not stay in that configuration for long; it will return to the original position as there will not be a force to keep it in the inverted state.

**Figure 3.**
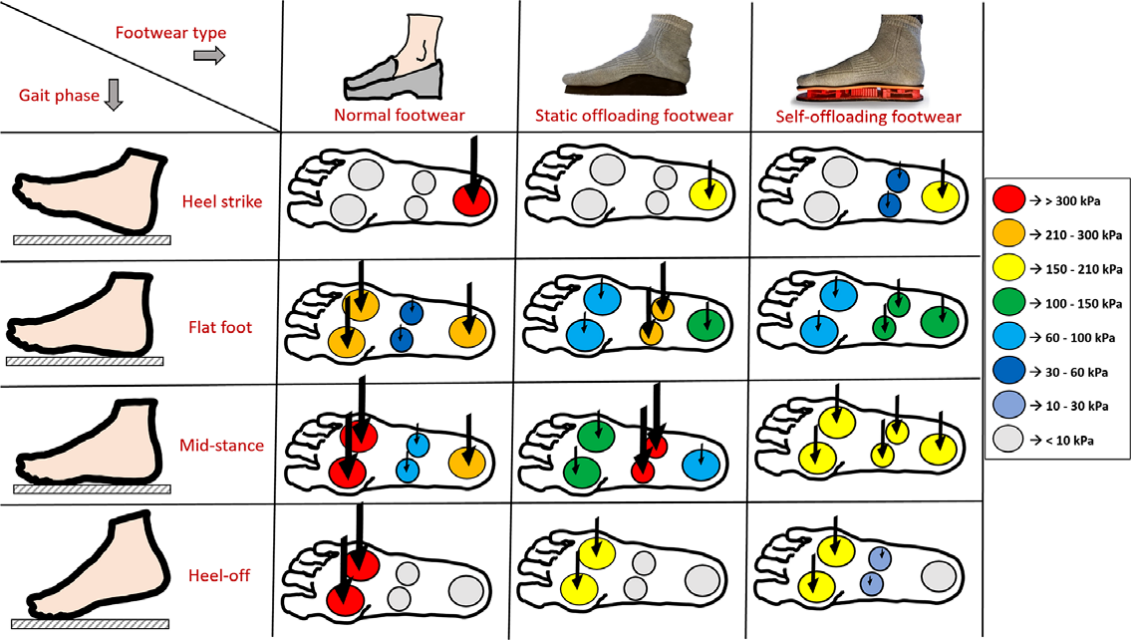
Comparison of normal footwear, static offloading footwear, and self-offloading footwear using snapping arches during four phases of the gait cycle, i.e., heel strike, flat foot, midstance, and heel off. The color code and the size of the arrow qualitatively indicate the pressure distribution on the plantar surface for all three types of footwear.

The advantage of self-offloading footwear over normal footwear and static offloading footwear is qualitatively compared for different gait phases in Figure 3. In the case of normal footwear, the plantar pressures at heel and forefoot regions exceed the maximum threshold values and are prone to ulceration if the same footwear is used for a long duration. These high-pressure regions are offloaded by using the static offloading footwear keeping the pressure below 200 kPa. However, usage of such footwear causes a high-pressure region to shift to the midfoot area, as stated earlier. On the contrary, in the case of dynamic self-offloading footwear, the arches placed inside the shoes snap to their inverted shape at a prescribed threshold force, such that no region would take pressure more than 200 kPa. Thus, the self-offloading footwear can reduce the risk of ulceration by redistributing pressure at the high-pressure regions.

The challenge in making the proposed concept of dynamic self-offloading is to design arches that snap at designated forces and return to their original configuration after a prescribed time. The details of this are discussed in section “Embodiment Design” after discussing materials and methods next.

## Materials and Methods

### Subjects

In our study, 100 subjects were recruited from the Department of Podiatry at KIER (Karnataka Institute of Endocrinology and Research). They were chosen based on criteria such as no history or presence of ulceration and foot amputation. Any severe complications to the feet were excluded from the study. The individuals fulfilling the criteria were examined by the doctor. They were informed about the study before participation, and written consent was obtained from all subjects.

### Data Collection

**Figure 4.**
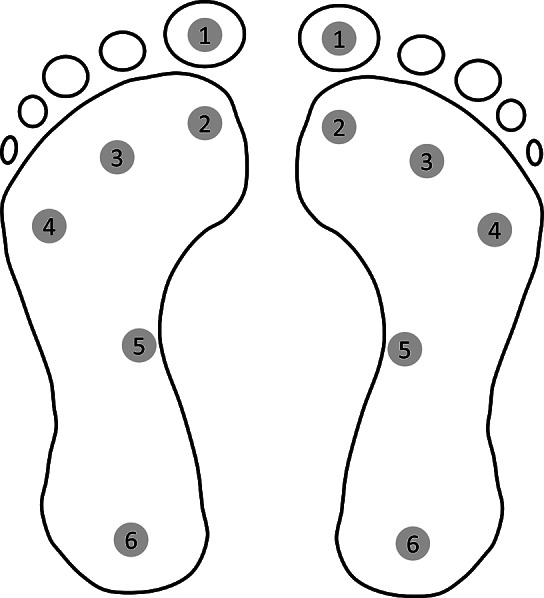
Test points for neuropathy assessment of feet (Source: KIER).

The height and body weight measurements were taken for each participating subject. Also, these subjects were required to undergo a series of tests which are common for all diabetic patients at KIER. The series of tests included in the study were neuropathy assessment test, barefoot pressure measurement, and in-shoe pressure measurements whenever required. The neuropathy was assessed by monofilament test, thermal thresholds, and vibration perception using the Bio-Thesiometer instrument. For all three tests, feet were divided into 12 test points as shown in Figure 4. A 10 g monofilament was used to check tactile sensation at the test points. Vibration detected below 15 V across all test points was considered as normal whereas above 25 V at any test point was considered as severe abnormality. A failure to perceive an increase and decrease in temperature up and down by 5°C for hot and cold perception from ambient temperature was considered as abnormal. Based on the results of these tests, subjects were categorized as neuropathic and nonneuropathic diabetics.

### Plantar Pressure Measurement

The emed by Novel pedography platform calibrated with capacitive sensors having a total of 1,760 sensors with two sensors per square centimeter was used to obtain the barefoot plantar pressure distribution while walking. The platform could measure pressure in the range of 10–1,270 kPa with an accuracy of ±7% and at a frequency of 25 Hz.

In-shoe pressure measurement was done using the Pedar-X measurement system by Novel. The data was collected using 1.9 mm-thick insoles available in different shoe sizes embedded with 99 sensors having approximately two sensors per sq. cm spatial resolution. An instructor from the hospital supervised the entire procedure to ensure that the subjects walked correctly.

### Data Analysis

From height and weight measurements of all recruited subjects, the BMI was calculated, and subjects were classified as per the standard BMI range, that is, BMI <18.5 were as underweight, 18.5 < BMI < 25 as normal, 25 < BMI < 28 as overweight, and above 28 as obese (Centers for Disease Control and Prevention, September, 2021). The data collected from the neuropathy assessment test was used to categorize the subjects as neuropathic and nonneuropathic diabetics for further analysis. The arch index, which quantifies the arch in the foot of the subjects, was calculated in accordance with the literature. Subjects with an arch index less than 0.21 were classified as high arch, arch index between 0.21 and 0.28 is considered as normal, and above that as a low arch (Periyasamy and Anand, 2013). The pedoscan reports were used for the analysis of pressure distribution on the plantar surface for all subjects.

**Figure 5.**
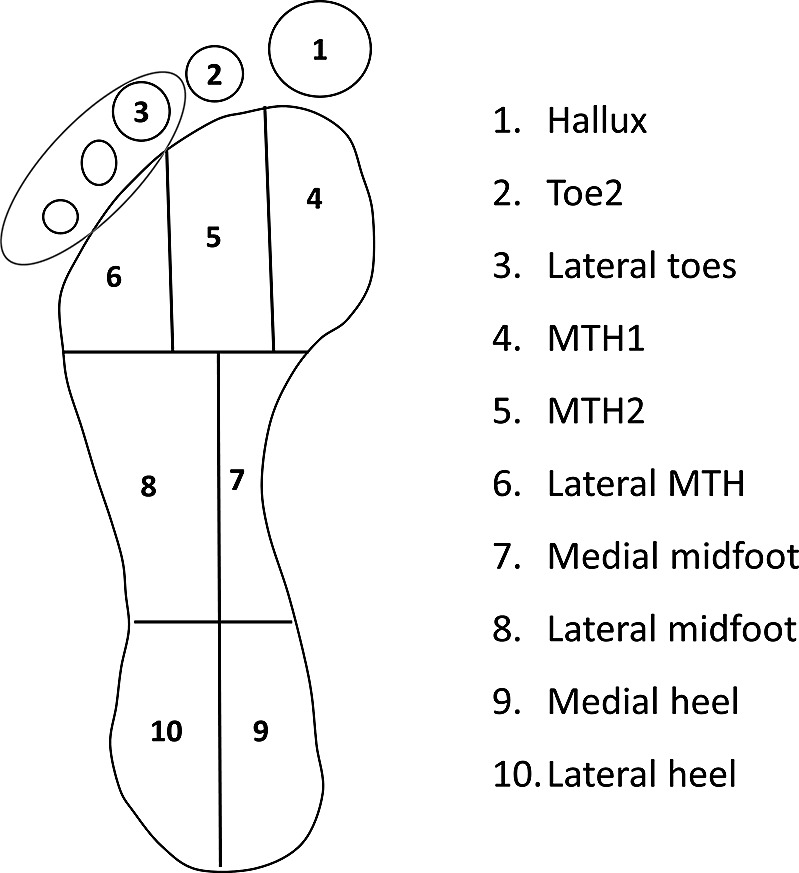
Foot division into ten anatomical regions.

The total area of the underside of the foot was divided into 10 anatomic regions, as shown in Figure 5 (Owings et al., 2008). The peak pressure was calculated for each region for each subject. Data were analyzed statistically using a Student’s *t*-test to check the variation of peak plantar pressure for neuropathic and nonneuropathic subjects.

### Results of Data Analysis

The demographic data for the subjects recruited in this study are given in Table 1. The duration of the disease showed a significant difference (*p* = .007) between neuropathic and nonneuropathic subjects. The BMI, arch index, and age did not show any significant difference between the two groups. The peak plantar pressure variation for 10 anatomical regions of the foot for two groups is shown in Table 2. In terms of pressure, at MTH1, pressure for neuropathic subjects differs significantly from other groups (*p* = .02). It is observed that the forefoot region and heel region bear maximum pressure for all subjects, and it is above the threshold value of 200 kPa.

**Table 1.** Demographic data: mean and standard deviation (SD) between two groups

Details	Neuropathic diabetics	Nonneuropathic diabetics	*p*-value
	Mean (SD)	Mean (SD)	
Number of subjects	71	29	
BMI (kg/m^2^)	26.54 (4.56)	26.4 (4.86)	.90
Age (years)	55 (10.99)	56.1 (8.71)	.52
Arch index	0.24 (0.11)	0.24 (0.07)	.79
Duration of diabetes (years)	9.82 (5.78)	6.3 (4.29)	.007^a^

a Represents statistically significant difference (*p* < .05, *t*-test).

**Table 2.** Plantar pressure distribution: mean and standard deviation (SD) between two groups

	Regions	Neuropathic diabetics	Nonneuropathic diabetics	*p*-value
		Mean (SD)	Mean (SD)	
Peak plantar pressure (kPa)	Hallux	257 (152.86)	229.04 (104.22)	.32
	Toe-2	128.62 (63.36)	131.54 (60.16)	.76
	Lateral toes	87.69 (46.90)	93.08 (62.48)	.72
	MTH1	221.54 (75.39)	190.96 (39.32)	.02^a^
	MTH2	286.31 (68.07)	290.00 (58.82)	.96
	Lateral MTH	274.69 (61.69)	284.81 (71.44)	.61
	Medial midfoot	109.55 (41.69)	125.77 (60.16)	.18
	Lateral midfoot	126.92 (51.67)	154.81 (70.90)	.10
	Medial heel	213.69 (50.30)	211.35 (47.26)	.60
	Lateral heel	208.08 (49.73)	210.19 (47.40)	.86

a Represents statistically significant difference (*p* < .05, *t*-test).

## Embodiment Design

### Force Calculation Using Image Processing

**Figure 6.**
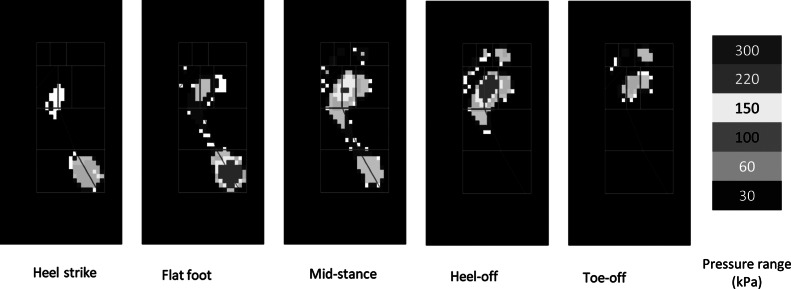
Pressure distribution during the gait cycle in ten anatomical regions of plantar surface.

For the self-offloading insole, we design the arches such that they snap at a threshold load and return to the original undeformed configuration from their inverted shape within a certain duration. Furthermore, in each region, we place an array of arches such that they can withstand the maximum load developed in that region during any phase of the walking cycle. They can be different for different regions depending on the real-time plantar pressure values. We analyze the plantar pressure at all the 10 regions in each gait phase to get the region-wise force distribution. Although the version of Novel software (to which we have access) provides the average pressure data for a complete gait cycle, obtaining region-wise pressure for each phase of the gait cycle requires some manual processing. Considering this, we used image processing to get the region-wise force distribution in each gait phase, which was further used for the placement of the arches at a particular foot region. For this purpose, we extract the real-time plantar pressure data for all the subjects recorded during barefoot walking. We capture the images of all the five phases of the gait cycle, that is, heel strike to toe-off. An in-house image processing code was used to read and extract the pressure value from the corresponding pixel values of the captured images. The code can read off the pressure values for normal as well as abnormal gaits. We divide the plantar surface into 10 standard regions, as shown in Figure 6. For different pressure ranges, we have different color bars, and each color has its own pixel value. As shown in Figure 6, each pressure value is represented by a square section, and each of these square sections represents the area of the sensor underneath the measuring surface. These pressure sensors are of the dimensions 0.7 cm 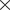 0.7 cm or an approximate area of 0.5 cm^2^.

**Figure 7.**
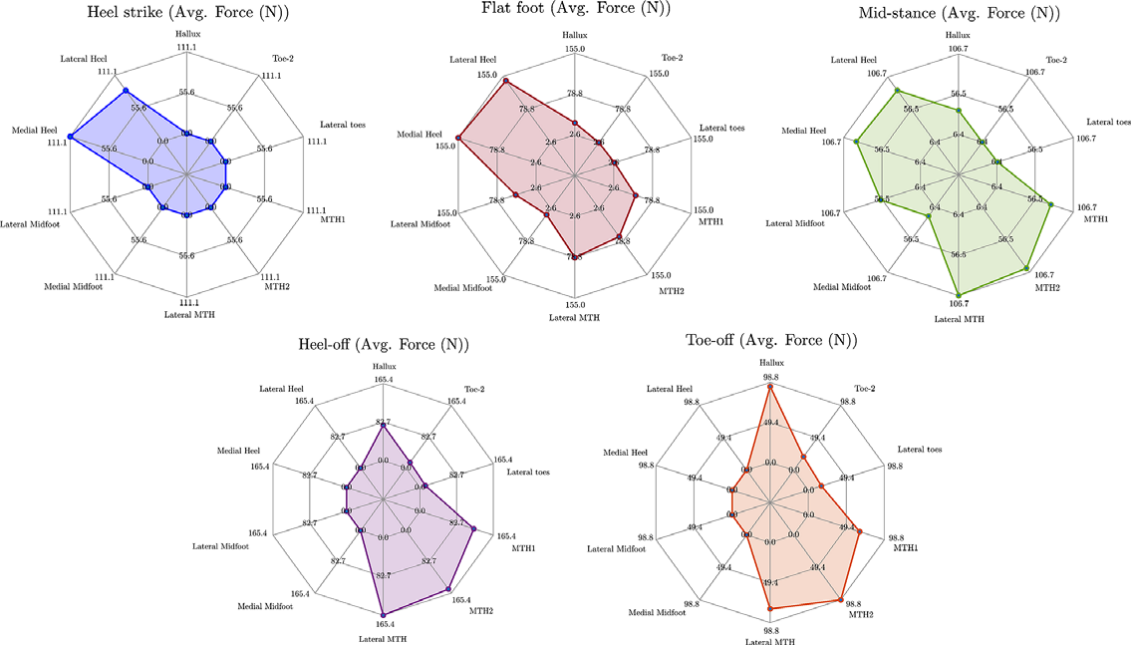
Average force distribution for gait cycle phases (weight up to 60 kg).

We calculate the number of pixels of each color present within a certain region and multiply it with the corresponding pressure value to get the approximate pressure for that region. We get the force value on each sensor using this approximate pressure value and the area of the sensor. We cumulate all these force values to get the total force for that certain region of the foot. We continue the same process for all 10 regions. Finally, we use these force values to decide the number of arches that should be placed in an array to bear the maximum load at that region. Also, we use these images and the real-time plantar pressure values to determine the time taken for each phase of the gait cycle. By knowing the phase timing, we design the arches for a particular switchback time such that after a certain region gets offloaded, the arches return to the original shape before the next gait phase starts. Figure 7 shows the average force distribution for each of the ten 10 anatomical regions for different phases of the gait cycle.

### Analysis of Snap-Through Arches

During the gait cycle, the contact area and the contact force between the sole and the ground change with time. At a certain region, the contact force varies abruptly and remains nonzero till that region is in contact with the ground surface. As discussed earlier, our aim is to reduce this contact force below a prescribed threshold value to avoid any foot complications. For that purpose, we use an array of cosine arches with fixed–fixed boundary conditions as the offloading element. Due to inherent nonlinearity, these arches snap to their inverted shape by changing their curvature once the applied force exceeds a certain limit. Eventually, the arch enters a negative stiffness region that helps to offload the high-pressure areas. These arches again switch back to their original as-fabricated shape once we remove the applied load. Hence, they are snap-through in nature, that is, once these arches snap, they do not need any actuation to return to their original shape. Therefore, to have an efficient self-offloading mechanism, we accurately tune the switching force and the switchback time (time taken by an arch to switch from the inverted shape to the original state) of these snapping arches.

**Figure 8.**
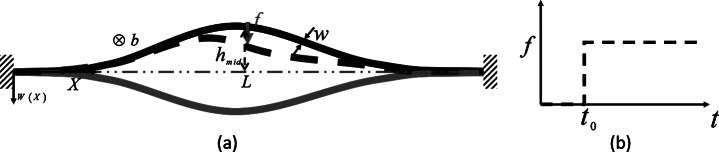
(a) A fixed-fixed arch with all the geometric parameters and a point load at the centre in three different configurations; as-fabricated (black curve), intermediate (blue dashed curve), and extreme inverted position (red curve) before returning to as-fabricated shape, and (b) a step load of magnitude is applied on the arch at time.

Let us take a fixed–fixed arch of the span *L*, the in-plane width *w*, the out-of-plane breadth *b*, the cross-sectional area 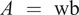 , the second moment of area 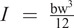 , and the mid-span height 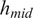 , made up of a material having Young’s modulus *E* and density 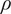 . There is a step load of magnitude *f* that is applied at the mid-span of the arch, as shown in Figure 8. We analyze this kind of snap-through arch when there is a step load in the form of a contact force applied to it. The maximum force or the switching force, *f_s_*, that the arch takes before it switches to its inverted shape is found as a solution of a quadratic polynomial [Maharana et al.] (see Supplementary Material for derivations) given by: 
(1)

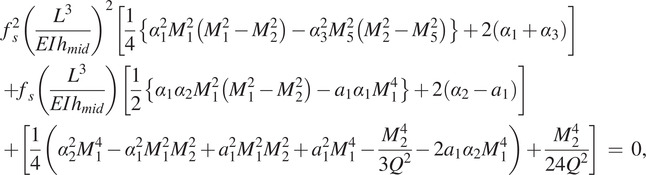

where 
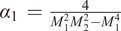
 , 
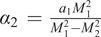
 , 
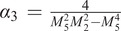
 , 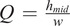 , 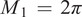 , 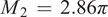 , and 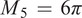 . For a specific value of the as-fabricated shape, 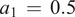 , the dynamic switching force ( 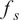 ) is expressed in terms of the arch geometric parameters as
(2)

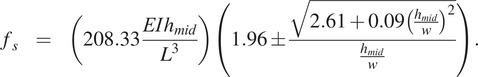


At this switching point, the asymmetric bifurcation or the second mode switching occurs, and the magnitude of the second mode weight becomes nonzero [Fung and Kaplan (1952), Palathingal and Ananthasuresh (2019), Qiu et al. (2005)]. Equations (1) and (2) give us two sets of switching force and second mode weight; one is real-valued, and the other is complex-valued. We choose the force magnitude corresponding to the real-valued second mode weight as the dynamic switching force of the arch. We use the analytical expression in Equation (2) to select the design parameters for the snap-through arch to customize the self-offloading insole based on the person’s body weight.

Once arches at a certain region deform and offload the region, they come up to their original shape before the next gait phase starts. Although the switchback occurs fast, it does take a finite duration of time. Hence, designing the arches for a particular switchback time based on the walking speed is important in our analysis. For instance, let us take the gait phase of the subject as 40 ms. If the switchback time of the arch is more than 40 ms, foot will progress to the next phase of gait, but arches in that area will come to their initial shape after some time. As the switchback is a fast phenomenon, it will apply a jerk to the foot, which may cause some complications. But if the switchback time is less than 40 ms, the arch comes up to its original shape along with the foot and aid for a smooth transition.

We define the switchback time of an arch as the time taken by the arch to return from the inverted state to its initial as-fabricated shape. Getting an explicit analytical solution for the switchback time does not seem possible when higher modes are considered. Hence, we simplify the dynamic equation in terms of the mid-point displacement, 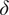 , to get the switchback time (see the Supplementary Material for the derivations) as
(3)

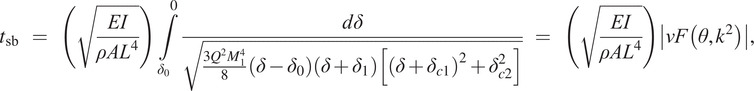

 where
(4)

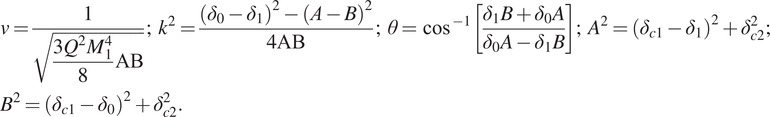

Here 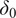 (mid-point displacement before switching back) and 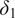 are the two real roots, and 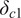 , 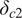 are the real and imaginary parts of the two complex roots of the polynomial function:
(5)

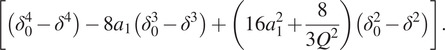

The two real roots signify two extreme positions of the free oscillations such that 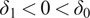 , that is, the two real roots lie on either side of the zero-deflection state, and 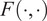 is the elliptic integral of first kind. For a specific arch shape, 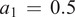 , the expression for the switchback time w.r.t. all the geometric parameters are calculated using Equation (3) and is expressed as:
(6)

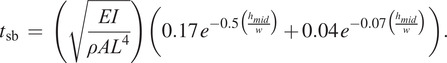

**Figure 9.**
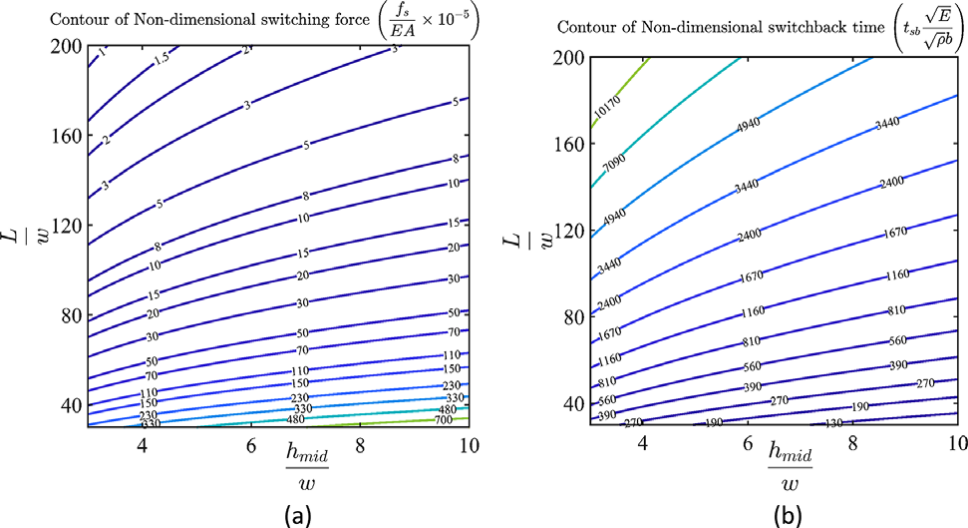
The contour of (a) dynamic switching force and (b) switchback time of a fixed-fixed arch for different arch geometries.

The contour of nondimensional switching force and switchback time of fixed–fixed arches for different nondimensional geometric parameters are shown in Figure 9. We use these figures to decide the arch geometries such that it satisfies the switching load and switchback time constraints. The design procedure of the self-offloading insole is discussed in the next section with a working prototype.

## Prototyping and Testing of Self-Offloading Footwear

### Design and Customization of Offloading Mechanism

We design and customize the self-offloading footwear based on the person’s body weight, plantar pressure, and walking speed using Equations (2) and (6). We design and arrange the arches such that the high plantar pressure regions are offloaded. The process of customization starts from the pedoscan done at KIER. As discussed earlier, we analyze the plantar pressure data and find the region-wise maximum plantar force for a gait cycle. We also get the walking speed of the patient from the real-time data available to us. First, we get the normalized switching force and the switchback time for a fixed *Q* value. Next, the mid-span height 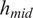 , the in-plane width *w*, the span *L*, and the out-of-plane breadth *b* are decided to get the actual switching force and switchback time of the arch. This selection of the arch dimensions is not unique, that is, we may get different combinations of geometric parameters for a given material property that results in the same switching force and the switchback time of the arch. They can be selected based on the availability of design space.

**Figure 10.**
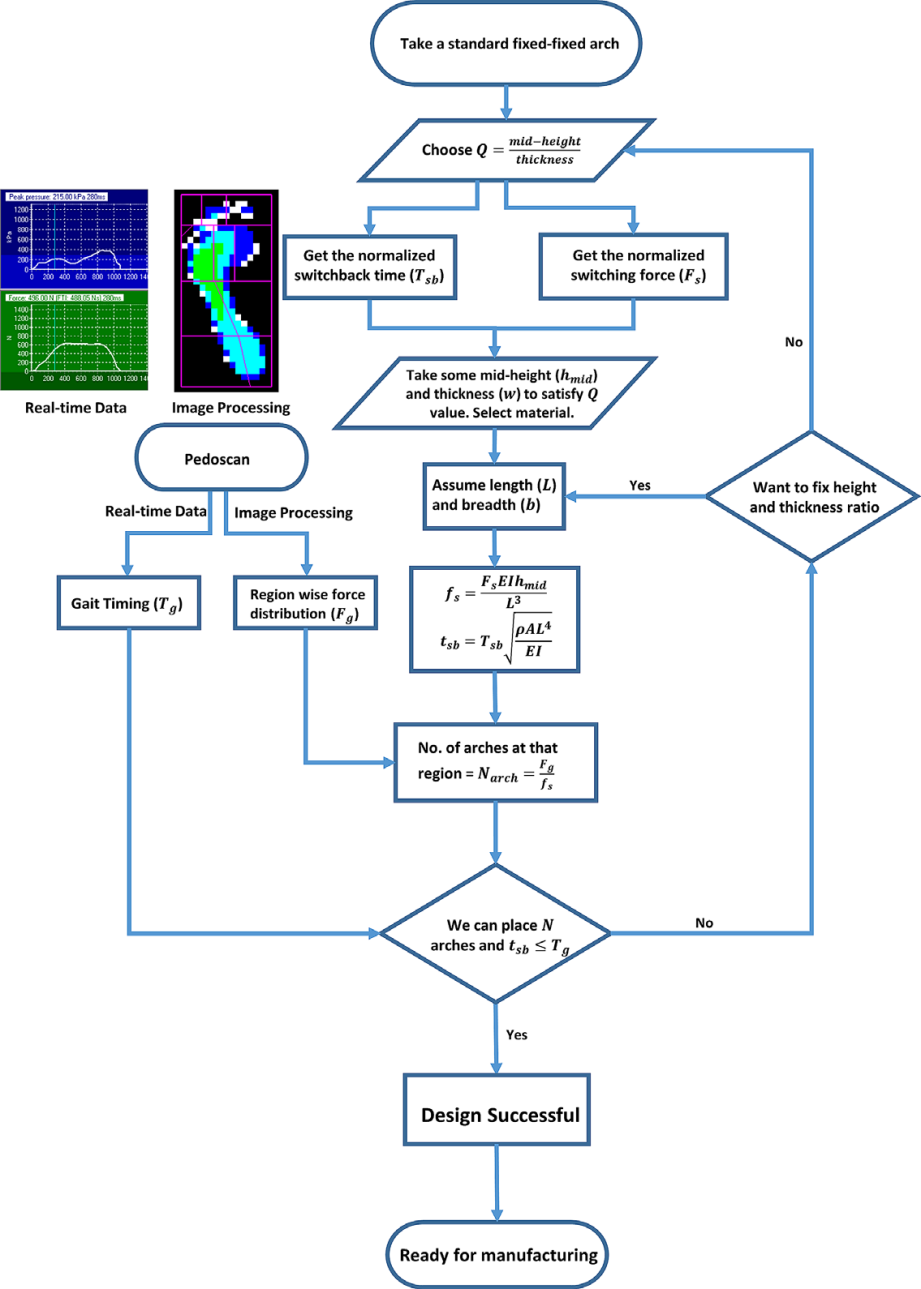
Flow chart to choose dimensions and the number of arches for plantar regions.

For designing the self-offloading insole, we know the maximum area available to place the arches together. We calculate the total number of arches, 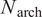 , from the known maximum force at each plantar region and the actual switching force of the individual arch. After deciding the number of arches, we recheck if those many numbers of arches can be placed in that confined region and the switchback time of the grouped arches is less than a person’s walking speed or not. Once both the conditions are satisfied, we finalize the dimensions of the arch, and the same process is followed for the other regions of the foot. The dimensions of the arch can be modified at multiple levels by changing *Q* and/or all other geometric parameters for the given foot area and switchback time. The complete procedure for designing the footwear is given in a flow chart (see Figure 10). Once the dimensions of the arches were decided, SolidWorks software was used to model the complete assembly of the insole with arches. The working prototype was made according to the given procedure and tested, details of which are described further.

### Prototyping

**Figure 11.**
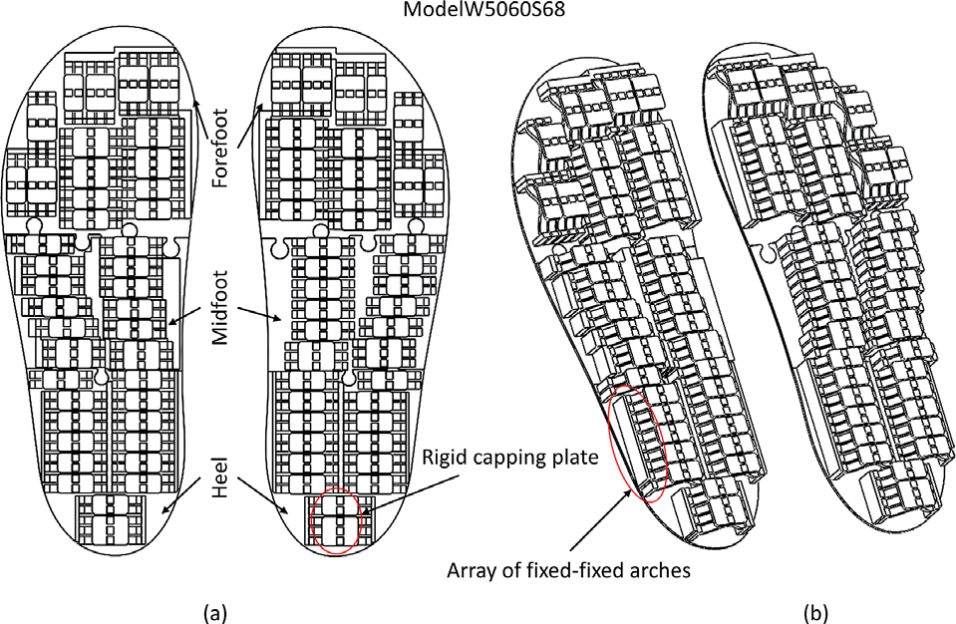
Arrangements of arches for weight 50-60 kg and insole size IN 6-8. (a) CAD model showing different regions of the prototype and the top rigid capping plate; (b) view from a different perspective showing the array of fixed-fixed arches.

The fixed–fixed snap-through arches were arranged in an array (see Figure 11) to take the load while walking. In order to ensure even load distribution among the arches and to move them together, they are held together using rigid capping plates. These capping plates also ensure that the foot rests evenly on arches along with balanced walking. As shown in Figure 11, the prototype was divided into three parts, namely forefoot, midfoot, and heel regions so that whenever there is a need for a replacement of any part from the insole, we can modify only that part instead of replacing the entire insole. It reduces the time needed for modification as well as for manufacturing the entire assembly. In a diabetic foot, the progression of weight distribution occurs from the heel to the forefoot and then to the toes. Subsequently, it results in multiple high-pressure areas, especially in the heel and forefoot. The reason for this is deranged proprioception and muscular nonequilibrium. The diabetic peripheral neuropathy and the abnormal glycation of ligaments and tendons make the plantar surface less elastic and malleable to the pressures during normal gait cycle. This results in an excessively supinated or a pronated foot causing abnormal high pressures at the heel as well as the forefoot region. Therefore, we place more arches in this region compared to the rest of the part of the foot. The initial design for the prototypes was developed, and one of them is shown in Figure 11. We design our models for different ranges of weight categories and foot sizes. For example, a subject having foot size 7 (inch) and weighing 55 kg during testing can use an insole Model W5060S68 (see Figure 11) designed for a weight category of 50–60 kg without worrying about the weight gain or loss. Similarly, the arches can be arranged to offload the high-pressure areas for different weights and foot sizes as per the requirements.

**Figure 12.**
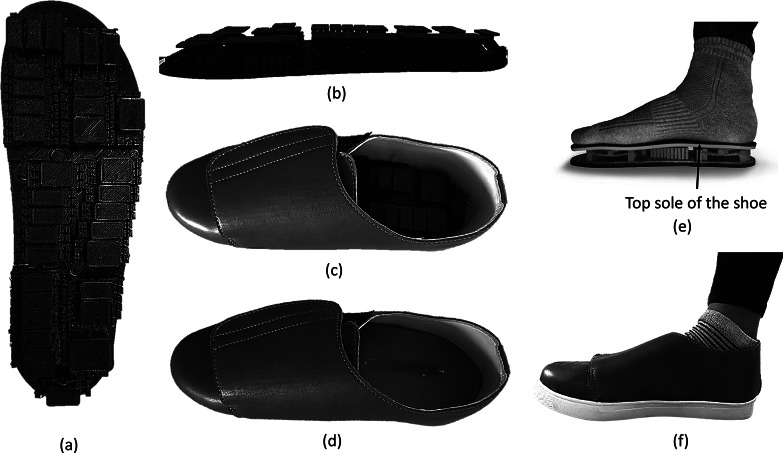
3D-printed prototype of self-offloading insole and customized footwear. (a) top view of the insole, (b) side view of the insole, (c) footwear with the self-offloading insole (d) footwear with the self-offloading insole underneath the top sole, (e) 3D-printed in-sole with two layers of soft sole on it to provide cushioning effect, and (f) one of the volunteers wearing the self-offloading footwear.

We used the fused deposition modeling (FDM) 3D-printing technology to fabricate the prototypes. This process involved selecting materials for 3D printing that are suitable for biomedical applications. We made a few samples with those materials and compared them for flexibility, specific strength, and longevity. We chose thermoplastic polyurethane (TPU) as it satisfied our need for the application. Figure 12 shows the complete prototype with 3D-printed insole.

### Mechanical Testing of Prototype

**Figure 13.**
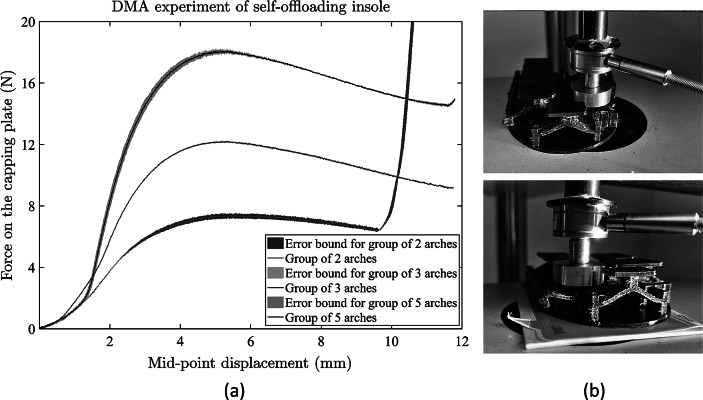
Mechanical testing of the self-offloading insole. (a) force-displacement plot of different groups of arches with standard deviation, (b) testing of the insole at different locations using the load cell.

The designed self-offloading insole was tested mechanically to check if the prototype helps in offloading by measuring the force-displacement graph, as shown in Figure 13a. These mechanical tests were performed using a Bose Electroforce 3200 (Bose Corp., Eden Prairie, MN) instrument equipped with two parallel compression platens (see Figure 13b). An unconfined displacement control tests at 0.1 mm/s were performed on different groups of arches (group of two arches, three arches, and five arches), and the reaction force from the capping plates was measured. Displacements were measured using a linear variable differential transformer (LVDT) and the forces from a load transducer (Bose Corp., ±22.5 N). The tests were performed three times on each group of arches until the displacement was two times the arch height or the load limit of the transducer (12 mm) reached.

### Clinical Testing of the Prototype

After 3D-printing the prototype, it was tested at KIER for a nondiabetic subject (52 kg, Female, foot size IN 6). First, the barefoot plantar pressure data is obtained. It showed plantar pressure above 200 kPa at forefoot and heel regions, hence chosen to check the offloading with prototypes. The 3D-printed self-offloading insole was placed in a regular shoe to check for the offloading. Before taking the actual data for both barefoot and in-shoe pressure, the subject was asked to walk freely so that a normal gait cycle was ensured. For each measurement, the subject was asked to walk three times, and data were recorded at each walking trial. The average pressure value of all three walking trials was considered to compare the results between barefoot and in-shoe measurements.

**Figure 14.**
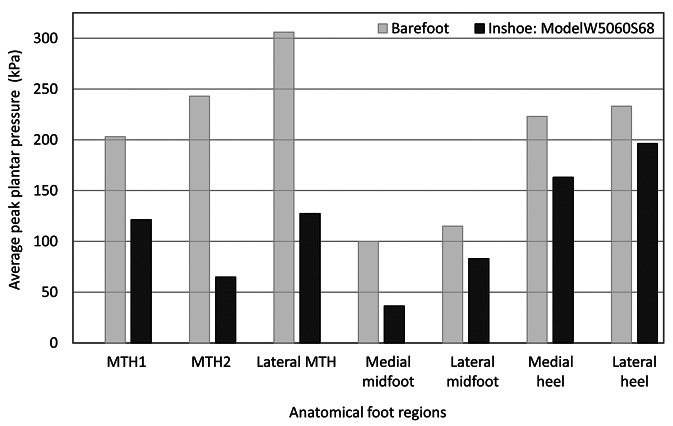
Average barefoot and in-shoe peak plantar pressure variation for anatomical foot regions.

The data collected from both barefoot and in-shoe pressure measurements were extracted from Novel software. The critical pressure value considered for offloading high-pressure regions is 200 kPa. The average peak plantar pressure obtained for barefoot and in-shoe with the prototype is shown in Figure 14. Model W5060S68, when walked with, showed offloading for all the 10-foot regions (see Figure 14). At the forefoot region, we observed a significant offloading of 57.34%, followed by midfoot and heel regions that showed offloading of 45.75 and 21.32%, respectively.

## Discussion

High plantar pressure in diabetic patients needs to be prevented to avoid further complications such as ulceration and foot amputation. Various methods in terms of offloading pressure are proposed and implemented from time to time to lessen the effects of high-plantar pressure. Pedography and in-shoe pressure measurement techniques provide investigators to analyze the real-time pressure and modify or develop better diabetic footwear. Offloading devices available in the market cannot provide relief to the foot for a long duration due to their own limitations, and mostly in static offloading, high-pressure areas keep changing. Hence, these devices need modifications or should be replaced frequently. This study is done to overcome the limitations of the static offloading method. The proposed self-offloading therapeutic footwear aims to provide pressure relief for diabetics to prevent complications.

The data analysis done in this study strengthened the observations from the literature. As the pressure varies from region to region in the foot, it was necessary to identify the at-risk areas and to determine the threshold forces required for the design of the footwear. As we are proposing this mechanism as a preventive method for reducing ulceration, we considered 200 kPa as critical pressure, and the rate of offloading success is compared according to this limit. Though the chances of developing an ulcer in nonneuropathic diabetics are less than neuropathic patients, we observed pressure above 200 kPa in nonneuropathies also. As the duration of disease showed a significant difference between these two groups of patients, we can design this self-offloading footwear for patients showing pressure above critical value as a preventive measure.

In this article, we have developed novel self-offloading footwear using nonlinear snap-through arches. We obtained a generalized closed-form solution for switching force and switchback time of a fixed–fixed arch. The nonlinear analysis is inherent in the buckling of slender arches and computationally intensive if finite element analysis is used. Consequently, it is time-consuming to design using trial and error finite element simulations. Therefore, the analytical expressions developed in section “Analysis of Snap-Through Arches” can be used to design the arches. For the self-offloading mechanism, the arches are designed in such a way that when one gait phase gets over, the arches underneath the sole return to their undeformed position to take the load in the next gait phase. The developed analytical expressions give us freedom to customize the footwear based on a person’s body weight and gait speed.

The mechanism presented in this study helped to offload the high-pressure regions that are above the critical value. The prototype is tested using the in-shoe method for dynamic offloading. The concept is showing positive results and can be implemented for reducing high plantar pressures. The entire foot sole is covered using an array of snap-through arches; therefore, it takes care of the pressure redistribution and maintains pressure below the critical limit while walking. The proposed mechanism shows offloading success validating this proof-of-concept prototype. It needs to be tested further for other targeted populations. Furthermore, it needs extensive testing on patients to study failure analysis of the product over an extended period.

## Notations

**length/span of the arch**
**in-plane thickness of the arch**
**out-of-plane breadth of the arch**
**cross-sectional area of the arch**
**second moment of area**
**Young’s modulus of arch material**
**density of arch material**
**arch height at**
**actual force applied on the arch at**
**nondimensional force applied on the arch at**
**time**
**time when force is applied on the arch**
**nondimensional time**
**force constant for nondimensionalization**
**time constant for nondimensionalization**
**a nondimensional geometric parameter**
**mode weight in the initial/as-fabricated shape of the arch**
**mode weight in the deformed shape of the arch**
**mode shape of the straight beam with fixed–fixed boundary condition**
**initial/undeformed/as-fabricated shape of the arch**
**deformed shape of the arch**
**number of mode shapes taken to capture the as-fabricated and deformed shape of the arch**
**strain energy due to bending**
**strain energy due to compression**
**work potential due to the external load**
**potential energy of the arch**
**kinetic energy of the arch**
**dynamic switching force of the arch**
**nondimensional dynamic switching force of the arch**
**deform mode weights of the arch before switching back**
**mid-height displacement of the arch**
**total potential energy of the arch before switching back**
**total initial energy stored in the arch before switching back**
**switchback time of the arch**
**nondimensional switchback time of the arch**
**elliptic integral of first kind**
**gait time of the subject**
**region-wise plantar force distribution of the subject**
**total number of arches at a particular foot region**

## Supplementary material

For supplementary material accompanying this paper visit https://doi.org/10.1017/wtc.2022.2. click here to view supplementary material
