# Supplementary material for: Self-offloading therapeutic footwear using compliant snap-through arches
Source: Wearable Technol. 2022 May 10;3:e7. doi: 10.1017/wtc.2022.2 (PMC10936371; doi:10.1017/wtc.2022.2)
Supplement: Supplementary file 1 [file S2631717622000020sup001.docx]

Self-offloading footwear using snapping arches

Maharana et al.

# Supplementary

We express the as-fabricated stress-free state, $h(x)$, and the deformed shape, $w(x)$, of the arches as a linear combination of the buckling mode weights of the corresponding straight beam as follows [21-23]:

(S1)

where $a_{i}$ and $A_{i}$ are the $i^{th}$ mode weights for the as-fabricated shape and deformed shape, respectively. Here $N$ is the number of modes that are taken for the approximation of deformed shape, and is the mode shape that satisfies the boundary conditions of the arch. For a fixed-fixed arch, the mode shapes are given as [23]:

(S2)

For a given as-fabricated shape, $a_{i}\text{s}$, and applied step load, $f$, the deformed mode weights, $A_{i}\text{s}$, are the function of time, $t$, and are determined by solving the dynamic equation of motion, which is derived next.

For an arch of Young’s modulus, $E$, and second moment of area, $I$, the bending strain energy is given by

$SE_{b}=\frac{1}{2}EI\int_{0}^{L} \left(\frac{d^{2}h}{dx^{2}}-\frac{d^{2}w}{dx^{2}}\right)^{2}dx$ (S3)

Due to the shallowness of the arch, i.e., and, we neglect the nonlinear terms in the bending energy expression. As the arch deforms in the transverse direction, it also undergoes axial compression (which is the primary source of nonlinearity), and the strain energy due to the compression is expressed as

$SE_{c}=\frac{1}{4}\frac{Ebw}{L}\left\{\int_{0}^{L_{1}} \left[\left(\frac{dh}{dx}\right)^{2}-\left(\frac{dw}{dx}\right)^{2}\right] dx\right\}^{2}$ (S4)

A point load, $f$, is applied at the mid-span length of the arch. The work potential due to the transverse load, $f$, at the mid-span length is given by

(S5)

The total potential energy of the arch includes the strain energy due to bending, compression, and the work potential, and is expressed as

$PE=SE_{b}+SE_{c}+WP$ (S6)

The kinetic energy of the arch is given as

$KE=\frac{1}{2}\int_{0}^{L} \rho A\left(\frac{dw}{dt}\right)^{2}dx$ (S7)

where $\rho$ is the material density of the arch. By using the Lagrangian method and substituting Eq. (S1) and (S2) in Eq. (S6) and (S7), we obtain the governing dynamic equations and the initial conditions of the arch as:

(S8)

where $F=\frac{f L^{3}}{EIh_{mid}}$ is the nondimensional step load applied at the middle of the arch, and represents the derivative w.r.t. the nondimensional time, $\tau=\sqrt{\frac{\rho AL^{4}}{EI}}$.

## Analysis for dynamic switching force

Obtaining the transient solutions from the dynamic equations of motion (Eq. (S8)) of the arch is difficult but is usually not needed for dynamic buckling analysis. What is required is the force value at which the arch enters to the negative stiffness region and switches to its inverted shape. As we neglect the damping of the arch, the system becomes conservative, and the total energy of the system has to be constant during the motion of the system. The value of the constant is determined from the initial conditions.

As the system is at rest initially, the total energy is equal to zero all the time. As the kinetic energy of the arch is a positive definite function of the velocity, in order to satisfy the principle of energy conservation, motion of the system is possible when the total potential energy is negative. As the potential energy of a stable equilibrium is negative, for a small magnitude of force, the arch oscillates about the stable equilibrium state (see Fig. S1 curve O-A). With sufficient increase in the force value, the oscillation amplitude increases, and the system reach an unstable equilibrium state. The total potential energy of the arch at this unstable equilibrium states change from positive to a negative value (see Fig. S1 curve O-B). At this unstable equilibrium point, with further force increment, the arch dynamically switches to its inverted shape (see Fig. S1 curve O-C). Therefore, the dynamic switching load for an arch corresponds to an unstable equilibrium state of zero potential energy [24-27].


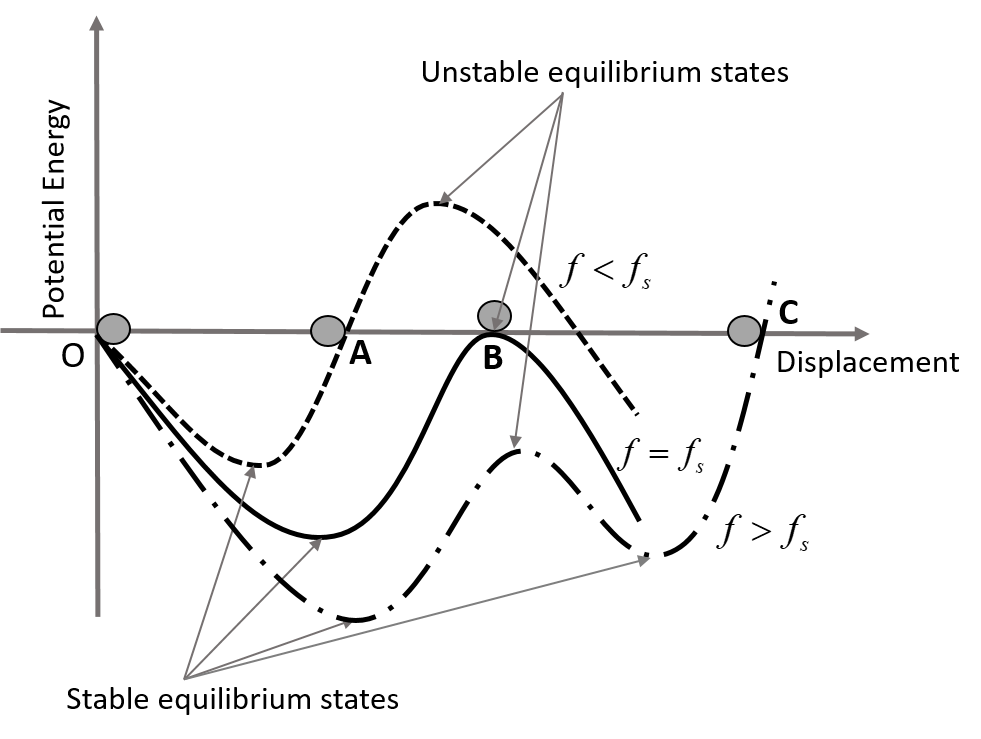


Fig. S1. Potential energy vs. displacement plot for a single degree of freedom system in three different loading cases; $f<f_{s}$ (curve O-A), $f=f_{s}$ (curve O-B), and $f>f_{s}$ (curve O-C). For $f<f_{s}$ the arch oscillates between O and A; for $f=f_{s}$ the arch oscillates between O and B, here point B corresponds to an unstable equilibrium state; and for $f>f_{s}$ the arch snaps to its inverted shape and oscillates between O and C.

Hence, to determine the switching load of the arch we solve the following set of equations by taking the first five modes in the deformed shape as

$PE=\frac{1}{4}\left(A_{1}^{2}M_{1}^{4}+A_{2}^{2}M_{2}^{4}+A_{5}^{2}M_{5}^{4}-a_{1}^{2}M_{1}^{4}+2a_{1}A_{1}M_{1}^{4}\right)+\frac{3}{8}Q^{2}C^{2}-2F\left(a_{1}-A_{1}-A_{5}\right)=0$ (S9)

$\frac{\partial PE}{\partial\partial A_{1}}=\frac{1}{2}(A_{1}M_{1}^{4}-a_{1}M_{1}^{4})-\frac{3}{2}Q^{2}CA_{1}M_{1}^{2}+2F=0$ (S10)

$\frac{\partial PE}{\partial A_{2}}=\frac{1}{2}(A_{2}M_{2}^{4})-\frac{3}{2}Q^{2}CA_{2}M_{2}^{2}=0$ (S11)

$\frac{\partial PE}{\partial A_{5}}=\frac{1}{2}(A_{5}M_{5}^{4})-\frac{3}{2}Q^{2}CA_{5}M_{5}^{2}+2F=0$ (S12)

where $C=a_{1}^{2}M_{1}^{2}-A_{1}^{2}M_{1}^{2}-A_{2}^{2}M_{2}^{2}-A_{5}^{2}M_{5}^{2}$ is the change in the arc length of the arch after the deformation. The third and fourth mode weights are not considered in the formulation of potential energy and equilibrium equations as those modes remain unaffected due to the nature of loading and give trivial zero solutions. After solving Eq. (S9) to Eq. (S12), the switching condition gets reduced to a quadratic equation in terms of the applied force, $F_{s}$, as:

(S13)

where $\alpha_{1}=\frac{4}{M_{1}^{2}M_{2}^{2}-M_{1}^{4}}$, $\alpha_{2}=\frac{a_{1}M_{1}^{2}}{M_{1}^{2}-M_{2}^{2}}$,$\alpha_{3}=\frac{4}{M_{5}^{2}M_{2}^{2}-M_{5}^{4}}$.

Due to asymmetric bifurcation, the second-mode switching occurs at the switching point and the magnitude of second mode weight, $A_{2}$, becomes non-zero at this state. The switching force equation (Eq. (S13)) gives us two sets of second mode weight ($A_{2}$), one of them is real-valued, and the other one becomes complex-valued. We choose the force magnitude corresponding to the real-valued second mode weight as the dynamic switching force of the arch. After getting the normalized switching force, we calculate the actual switching force ($f_{s}$) from the geometric parameters as:

$f_{s}=F_{s}\left(\frac{EIh_{mid}}{L^{3}}\right)$ (S14)

## Analysis for switchback time

The first mode is the primary mode that defines the deformed geometry of the arch at any instant of time. Therefore, there is no loss of generality in finding the switchback time by considering only the first mode of the deformed profile of the arch. By assuming that the arch is settled down at its inverted shape for a long time before switching back, we can take the initial velocity of the arch as zero. Although there is no external force acting on the arch, the arch moves to its as-fabricated shape by releasing the stored deformed-state potential energy [28]. Hence, for a single-mode approximation, Eq. (S8) is reduced to

(S15)

where $A_{1}^{0}$ is the initial deformed shape of the arch and represents the derivative w.r.t. normalized time $\tau$. By substituting $\delta=2\left(a_{1}-A_{1}\right)$, that signifies the mid-point displacement of the arch, in Eq. (S8), we get the equation in $\delta$ as:

(S16)

After multiplying both sides of Eq. (S16) with and integrating, we get

(S17)

where $E_{0}$ is the integration constant that depends on the initial conditions of the arch. Herein,$E_{0}$ only consist of potential energy and is expressed as:

(S18)

Eq. (S17) is the energy integral of the arch undergoing free oscillation. The total energy of the arch is conserved, and it is equivalent to a particle moving on a frictionless potential energy surface described by

(S19)

The time taken by the arch to move from the initial potential energy to zero-potential energy at $\delta=0$ is determined by integrating Eq. (S17) using Eq. (S18). Therefore, the nondimensionalized switchback time of the arch, $T_{sb}$, is given by

$\int_{0}^{T_{sb}} d\tau=T_{sb}=\int_{\delta_{0}}^{0} \frac{d\delta}{\sqrt{2\left(PE_{0}-PE\right)}}$ (S20)

The energy surface described by Eq. (S19) consists of one hill (maximum) and two valleys (minima). The valley corresponds to the stable equilibrium state, whereas the hill is an unstable equilibrium state. For an arch to switch back to its as-fabricated shape, the total initial energy should be greater than the potential energy associated with the hill of the surface. Therefore, for such a potential energy level, the surface $PE_{0}-PE$ has only two real roots between which the arch oscillates, passing through the zero-displacement point. For such an oscillation, the switchback time, $T_{sb}$, is determined from Eq. (S20) using the elliptic integral of first kind as:

(S21)

where

(S22)

The actual switchback time ($t_{sb}$) is expressed as:

$t_{sb}=T_{sb}\left(\sqrt{\frac{\rho AL^{4}}{EI}}\right)$ (S23)
